# Effect of γ-Heptalactone on the Morphology and Production of Monascus Pigments and Monacolin K in *Monascus purpureus*

**DOI:** 10.3390/jof8020179

**Published:** 2022-02-11

**Authors:** Ruoyu Shi, Qiaoqiao Luo, Yutong Liu, Wei Chen, Chengtao Wang

**Affiliations:** 1 Beijing Advanced Innovation Center for Food Nutrition and Human Health, Beijing Engineering and Technology Research Center of Food Additives, Beijing Technology & Business University (BTBU), Beijing 100048, China; shiruoy@126.com (R.S.); luoqiaoqiao7469@163.com (Q.L.); 18742058299@163.com (Y.L.); 2 Yunnan Plateau Characteristic Agricultural Industry Research Institute, Yunnan Agricultural University, Kunming 650201, China

**Keywords:** *Monascus purpureus*, γ-heptalactone, Monascus pigments, monacolin K, mycelial morphology

## Abstract

*Monascus* is used widely in Asian countries and produces various biologically active metabolites, such as Monascus pigments (MPs) and monacolin K (MK). In this study, the effect of γ-heptalactone on secondary metabolites and mycelial growth during *Monascus purpureus* M1 fermentation was investigated. After the addition of 50 μM γ-heptalactone, the yields of MPs (yellow, orange, and red) reached maxima, increased by 115.70, 141.52, and 100.88%, respectively. The 25 μM γ-heptalactone groups showed the highest yield of MK was increased by 62.38% compared with that of the control. Gene expression analysis showed that the relative expression levels of MPs synthesis genes (*MpPKS5*, *MpFasA2*, *mppB*, *mppC*, *mppD*, *mppG*, *mpp7*, and *mppR1*/*R2*) were significantly upregulated after γ-heptalactone treatment. The relative expression levels of MK synthesis genes (*mokA*, *mokC*, *mokE*, *mokH,* and *mokI*) were significantly affected. The mycelium samples treated with γ-heptalactone exhibited more folds and swelling than that in the samples of the control group. This study confirmed that the addition of γ-heptalactone has the potential to induce yields of MPs and MK, and promote the expression of biosynthesis genes, which may be related to the transformation of mycelial morphology in *M. purpureus*.

## 1. Introduction

*Monascus* is a filamentous fungus that has been extensively utilized to color or preserve food and medicine for thousands of years in Southeast Asian countries [1,2]. *Monascus* can be fermented to produce various functional metabolites, such as Monascus pigments (MPs), monacolin K (MK), γ-aminobutyric acid, ergosterol, enzymes, and organic acids, which are widely used in the food, nutrition, health care, brewing, and medicine industries [3,4,5,6].

MPs can be divided into three broad categories, namely yellow, orange, and red [7]. As natural and safe colorants, MPs can be used as functional food ingredients. They provide good coloring effects; can be easily biosynthesized [8,9]; and possess biological activities such as antioxidant, antibacterial, anti-inflammatory, and antitumor properties [10,11,12,13,14,15]. Therefore, MPs are widely used in the food, medicine, and cosmetics industries, and have good application prospects in the textile, printing, and dyeing industries [16,17,18,19]. *Monascus purpureus* and *Monascus rubbers* are the two main pigment-producing strains [20,21,22]. MPs are derived from the fermentation product of *Monascus*, composed of a polyketone chromophore and medium long-chain fatty acids. At present, six alcohol-soluble pigments (ankaflavin, monascin, monascorubin, rubropunctatin, monascorubramine, and rubropunctamine), which have similar structures, have been identified as the principal pigments of *Monascus* metabolism and have similar structures [23,24].

MK is a physiologically active polyketone that was first isolated from a culture of *M. ruber* by Endo in 1979 [25], and is also known as lovastatin in *Aspergillus terreus* [26]. MK is employed as an inhibitor of cholesterol biosynthesis, as it has a similar structure to 3-hydroxy-3-methyl glutaryl coenzyme A (HMG-CoA), and can inhibit HMG-CoA reductase activity [27]. MK has been used to regulate blood pressure and blood lipids, and can be developed as a lipid-lowering drug [28,29,30]. In 2002, Manzoni et al. determined the biosynthetic pathway of lovastatin in *A. terreus* [31]. Lovastatin biosynthesis is initialized by the catalyzation of lovastatin nine peptide synthase (LNKS) with acetyl CoA and malonyl CoA as the substrates [32]. In 2008, nine genes, termed *mokA*–*mokI*, were found in *Monascus pilosus*, which were highly analogous to the genes (*lovB*–*lovI*) of the lovastatin biosynthetic pathway found in *A. terreus* [33].

Current studies on MPs and MK have mainly focused on improving the yield. Research on MPs and MK production by *Monascus* submerged fermentation has mainly focused on improving metabolism by optimizing culture conditions and medium components, such as the inoculation amount, temperature, initial pH value, culture time, oxygen concentration, and nutrients [16,34,35,36,37]. Studies have also been performed under conditions such as high salt or sugar stress, and in a low-frequency magnetic field [38,39,40]. In addition to optimizing the culture medium and fermentation conditions, the biosynthesis of secondary metabolites of *Monascus* was also promoted by adding exogenous substances, such as glutamate, surfactants, ethanol, rutin, troxerutin, α-glucose rutin, ammonium nitrate, linoleic acid, and cAMP [41,42,43,44,45,46,47].

Lactones are a group of organic compounds characterized by lactone rings. Lactone-containing compounds are widely used as the main components of industrial fragrances [48]. Lactones also participate in various metabolic pathways owing to their antioxidant, antibacterial, and anticancer activities [49]. Moreover, γ-heptalactone induces and enhances penicillin production by shortening the lag period of *Aspergillus nidulans*, and increasing mycelial branching and spore formation [50]. Previous studies have illustrated that butyrolactone I was found to increase mycelial branching and spore formation, and promote the production of the secondary metabolite lovastatin in *A. terreus* [51]. In addition, butyrolactone I has been reported to induce a role in lovastatin biosynthesis [52]. Raina [52] and Palonen EK [53] believed that butyrolactone I positively regulated the expression of the global regulator gene *laeA* in *A. terreus,* and promoted conidiogenesis and lovastatin biosynthesis. 

γ-Heptalactone and butyrolactones are signaling molecules that affect the secondary metabolism and mycelial growth in the filamentous fungi *A. nidulans* and *A. terreus*. Previous studies have shown that lactones affect the production of lovastatin in *A. nidulans*, but the effect of lactones on *Monascus* has not been reported. This study aimed to determine whether lactones act as signaling molecules in the secondary metabolites of *Monascus*.

In the present study, the effect of γ-heptalactone on the biosynthesis of secondary metabolites in *M. purpureus* was investigated. The effect of different γ-heptalactone concentrations (0, 25, 50, and 100 μM) on the yield of MPs and MK was studied during *M. purpureus* fermentation. Moreover, the expression levels of MPs and MK biosynthesis genes and asexual development genes (*brlA*, *wetA*, and *laeA*) were analyzed to reveal the potential mechanism using real-time quantitative polymerase chain reaction (RT-qPCR). The morphology of mycelia and spores was observed by using a light microscope and scanning electron microscope (SEM), respectively. This study was mainly aimed at investigating the regulatory effect of γ-heptalactone on MPs and MK biosynthesis, the change in morphology and sporulation, and the improvement of the final yield of desired metabolites in *M. purpureus*.

## 2. Materials and Methods

### 2.1. Microorganism and Media

The *M.* *purpureus* M1 strain (CGMCC 3.0568) preserved in the laboratory was selected for this study. The strain was activated on the potato dextrose agar (PDA) medium (20 g/L glucose, 3 g/L peptone, 4 g/L yeast, 20 g/L malt, 20 g/L agar, 2 g/L KH_2_PO_4_, 2 g/L NaNO_3_, and 1 g/L MgSO_4_·7H_2_O) at 30 °C for 5 days [54]. The spore suspension (10^6^/mL) was prepared and inoculated into 50 mL of seed liquid medium (30 g/L glucose, 15 g/L soybean meal, 10 g/L peptone, 70 mL/L glycerol, 2 g/L KH_2_PO_4_, 2 g/L NaNO_3_, and 1 g/L MgSO_4_·7H_2_O) with 10% volume of inoculum. The seed culture was kept at 30 °C and 200 rpm (HZQ-F160, China) for 48 h in a 250-mL Erlenmeyer flask (This culture was nominated as the seed culture) [54,55].

### 2.2. Production of MPs and MK

The seed culture (10% *v*/*v*) was used to inoculate the MPs and MK production medium (20 g/L rice powder, 1 g/L MgSO_4_·7H_2_O, 2 g/L ZnSO_4_·7H_2_O, 2.5 g/L KH_2_PO_4_, 90 g/L glycerol, 5 g/L NaNO_3_, and 10 g/L peptones). Then, the inoculated production medium was cultured at 30 °C, at 200 rpm for 2 days. After that, the culture was incubated at 25 °C, at 150 rpm for 13 days [54,55]. Experimental lactone groups of γ-heptalactone, γ-butyrolactone, γ-caprolactone, γ-valerolactone, and γ-octalactone were added to the fermentation medium with 100 μM concentration. γ-heptalactone was added to the fermentation medium at different concentrations (0, 25, 50, and 100 μM) on the second day to identify the optimal concentration.

### 2.3. Determination of MPs

The analysis method of *M. purpureus* pigments was modified according to Chen et al. [56,57]. The fermentation broths were extracted with 70% ethanol, soaked in a water bath at 60 °C for 1 h in dark, and then filtered. Then, the absorbance at 505, 448, and 410 nm were determined. The color values of the pigments were obtained by multiplying the absorbance value with the dilution factor [58].

Yellow pigment color value (U/mL) = OD at 410 × dilution factor

Orange pigment color value (U/mL) = OD at 448 × dilution factor

Red pigment color value (U/mL) = OD at 505 × dilution factor

### 2.4. HPLC Analysis of MK Production

To determine the yield of MK, the fermentation broth was mixed with 70% methanol, and 30 min sonication treatment (250 W, 40 kHz) was conducted to extract MK. After centrifugation at 8000 g for 5 min, the supernatant was filtered through a 0.22 μm membrane for detection by HPLC (LC-20AT, Shimadzu, Kyoto, Japan) [59].

HPLC analysis was performed under the following conditions: Inertsil ODS-3 C18 column (150 mm×4.6 mm×5 μm), mobile phase (0.1% H_3_PO_4_: methanol = 35:65, *v*/*v*), flow rate of 1 mL/min, column temperature of 30 °C, an ultraviolet detector detected the wavelength at 237 nm, and an injection volume of 10 μL [60].

### 2.5. Microscopy Analysis of Monascus Mycelia and Spore

The growth and development of the mycelium and spore morphology were observed by using a light microscope (CX43, Olympus, Japan) and scanning electron microscope (SEM; Su8020, Hitachi Ltd., Tokyo, Japan). To observe the effect of γ-heptalactone on mycelial growth, 10 μL spore suspension was inoculated on PDA and was cultured at 30 °C, and the morphology was observed with a microscope. For the SEM analysis, the mycelium was fixed in a 2.5% glutaraldehyde solution for 12 h and washed twice with 0.1 M phosphate buffer (pH 7.2). Then, the mycelium was dehydrated with different concentrations of ethanol solution (30, 50, 70, 80, 90, and 100%) and each was repeated twice. The mycelium was collected after centrifugation at 12,000 rpm for 5 min and the supernatant was discarded. Then, the solvent hexamethyldisilazane (HMDS) was added and the sample was dried in the oven at 60 °C until it became powder [55]. The mycelial sample was observed using a Su8020 SEM (Hitachi Ltd., Tokyo, Japan).

### 2.6. Real-Time Quantitative PCR Analysis of MPs and MK Biosynthesis-Related Genes

Gene expression analysis was performed by RT-qPCR according to the methods of Zhang and Yang [54,55]. The total RNA from the mycelium was extracted by the polysaccharide polyphenol plant total RNA extraction Kit (Tiangen, Beijing, China). The first-strand cDNA was synthesized with a FastQuant RT Kit (with gDNase) (Tiangen, Beijing, China) and the SuperReal PreMix Plus (SYBR Green) kit (Tiangen, Beijing, China). RT-qPCR was accomplished using a CFX96 Real-Time PCR detection system (Bio-Rad, Hercules, CA, USA). The amplification cycle was as follows: 95 °C for 15 min, followed by a three-step PCR (40 cycles of denaturation at 95 °C for 10 s, annealing at 52 °C for 20 s, and extension at 72 °C for 30 s) [60,61]. The levels of relative expression were calculated by the 2^−^^△△Ct^ method. All values were normalized using the transcription level of the *GAPDH* gene. RT-qPCR was conducted in triplicate for each sample. Primers for MPs biosynthesis genes (GenBank accession no. MK764693.1), *mokA*-*mokI* (GenBank accession no. DQ176595.1), and *GAPDH* (GenBank accession no. HQ123044.1) were designed by Beacon Primer Premier 5 software, as shown in Table 1.

### 2.7. Statistical Analysis

Each experiment was repeated at least thrice. All statistical analyses were performed by one-way analysis of variance (ANOVA) using GraphPad prime 9.0. Numerical data were expressed as mean ± SD, and *p*-values <0.05 and <0.01 were considered statistically significant.

## 3. Results

### 3.1. Effect of Lactones on Secondary Metabolites in M. purpureus

**Figure 1:**
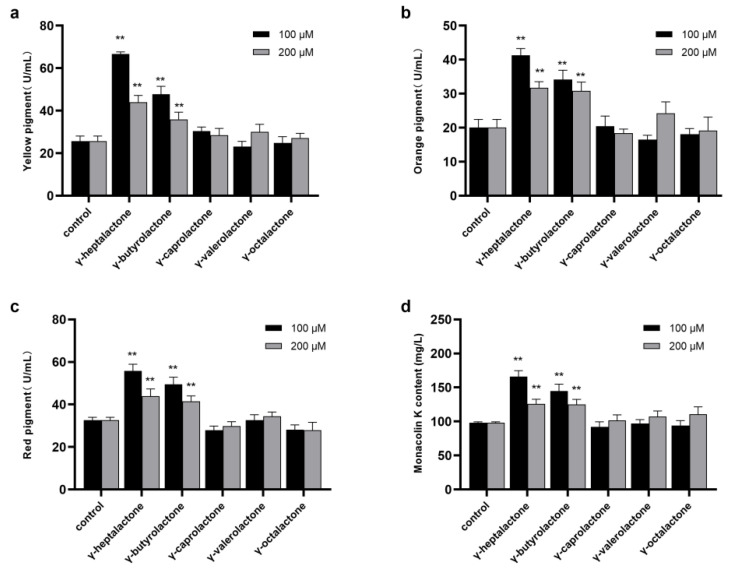
Effect of five lactones (γ-heptalactone, γ-butyrolactone, γ-caprolactone, γ-valerolactone, and γ-octalactone) on the MPs and MK synthesis in *M.* *purpureus* M1. (**a**) Yellow pigment, (**b**) orange pigment, (**c**) red pigment, and (**d**) MK. Data are expressed as the mean ± SD (*n* = 3). ** *p* < 0.01, compared to the control.

The effects of exogenously added lactones (γ-heptalactone, γ-butyrolactone, γ-caprolactone, γ-valerolactone, and γ-octalactone) on the biosynthesis of secondary metabolites are shown in Figure 1. As shown in Figure 1a–c, the maximum yields of MPs were higher in the γ-heptalactone and γ-butyrolactone groups than in the control group. The yield of MK was significantly promoted in the γ-heptalactone and γ-butyrolactone groups (*p* < 0.01) compared with the control group, while other substances showed no such effect (Figure 1d).

### 3.2. Effect of γ-Heptalactone on the Yield of MPs and MK in M. purpureus

**Figure 2:**
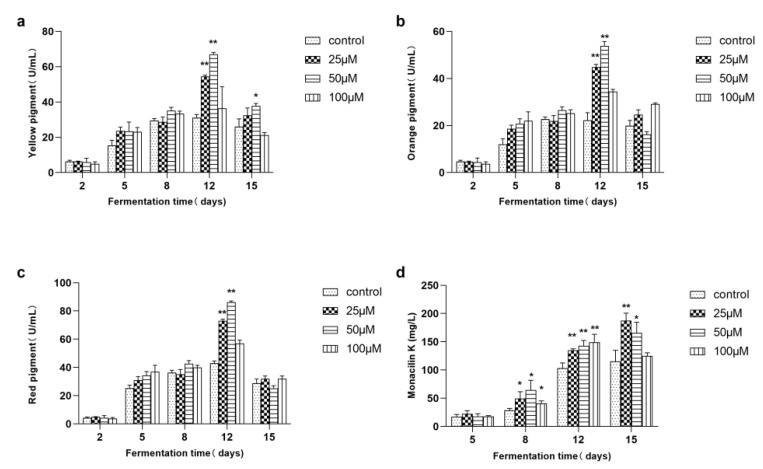
Effect of different γ-heptalactone concentrations on the yield of MPs and MK in *M. purpureus* M1. (**a**) Yellow pigment, (**b**) orange pigment; (**c**) red pigment, and (**d**) MK. Data are expressed as the mean ± SD (*n* = 3). * *p* < 0.05 and ** *p* < 0.01, compared to the control.

After the addition of γ-heptalactone, the yields of MPs and MK were analyzed throughout the fermentation process (Figure 2). The yields of yellow, orange, and red pigments at different culture periods (2, 5, 8, 12, and 15 days) were detected by spectrophotometry (Figure 2a–c). With the increase in γ-heptalactone concentration, the yield of MPs increased at first, but finally dropped to the same level as that in the control group. Although pigment production during the initial period (0–2 days) did not change, the addition of 25 and 50 μM γ-heptalactone resulted in higher pigment production after 5 days of incubation. The changes in the yields of the three pigments were generally consistent. In particular, the most significant increase was observed for the 25 and 50 μM γ-heptalactone treatment (*p* < 0.01). On the twelfth day, the MPs yield reached maximum level with γ-heptalactone doses of 50 μM, and the yield of yellow pigment, orange pigment, and red pigment was 115.70, 141.52, and 100.88%, respectively, higher than that of the control group, reaching 67.05 U/mL, 53.83 U/mL, and 86.25 U/mL, respectively. The MK yield was gradually stimulated with the γ-heptalactone treatment compared with that in the control from day 8 to day 15 (Figure 2d). The 25 and 50 μM γ-heptalactone groups showed the highest MK production on day 12, which was increased by 62.38 and 43.82%, respectively, compared with the control group. However, when 100 μM γ-heptalactone was added, the yield of MK reached its peak on day 12 and then dropped on day 15. The maximum yield of MK was achieved after 15 days of culture with 25 μM of lactone, which was 62.38% higher than that in the control group.

### 3.3. Effect of γ-Heptalactone on the Expression of MPs Biosynthesis-Related Genes

**Figure 3:**
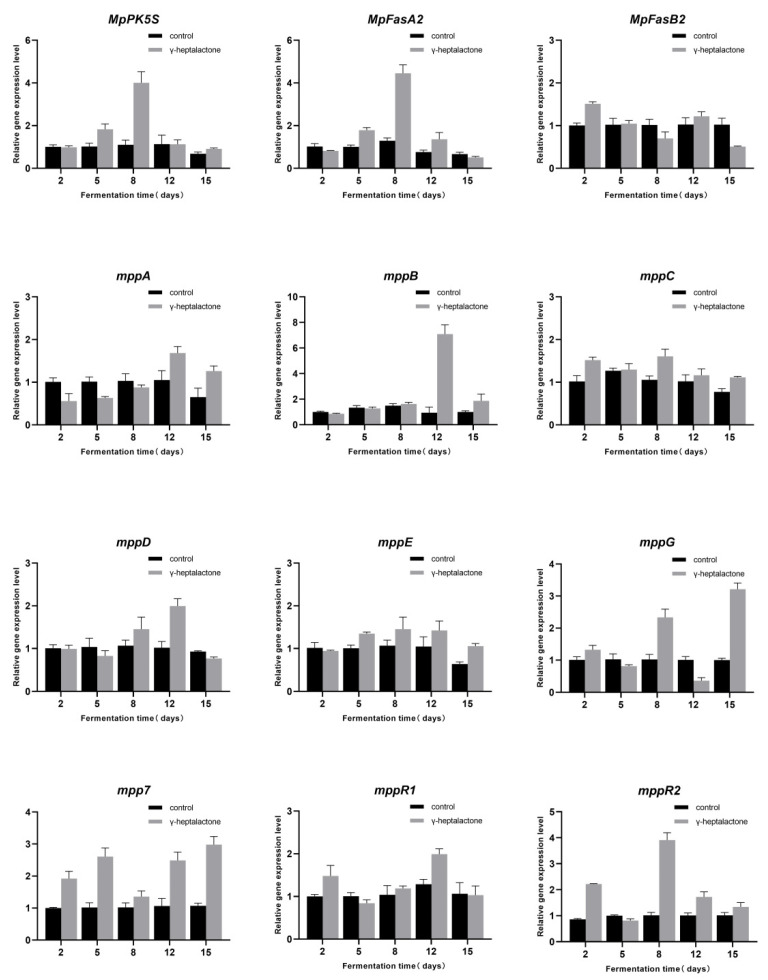
Effect of γ-heptalactone on the relative gene expression levels of MPs biosynthesis genes.

To investigate the effects of γ-heptalactone on MPs biosynthesis, we measured the expression levels of *MpPKS5*, *MpFasA2*, *MpFasB2*, *mppA*, *mppB*, *mppC*, *mppD*, *mppE*, *mppG*, *mpp7*, *mppR1*, and *mppR2* genes using RT-qPCR. As shown in Figure 3, the relative levels of *MpPKS5*, *MpFasA2*, *mppC*, and *mppE* genes were upregulated by 3.64-, 3.44-, 1.52-, and 1.36-fold, respectively, compared with the control group on day 8. On day 12, the expression levels of the *mppA*, *mppB*, and *mppD* genes increased by 1.94-, 7.51-, and 1.96-fold, respectively. The expression levels of *mpp7* and *mppG* were upregulated by 2.79- and 3.21-fold on day 15, respectively, whereas the expression levels of *MpFasB2*, *mppA*, and *mppE* did not change. The expression levels of two transcription factors *MppR1* and *MppR2* were increased by 1.55- and 3.86-fold, respectively, compared to that in the control.

### 3.4. Effect of γ-Heptalactone on the Expression of MK Biosynthetic Gene Cluster

**Figure 4:**
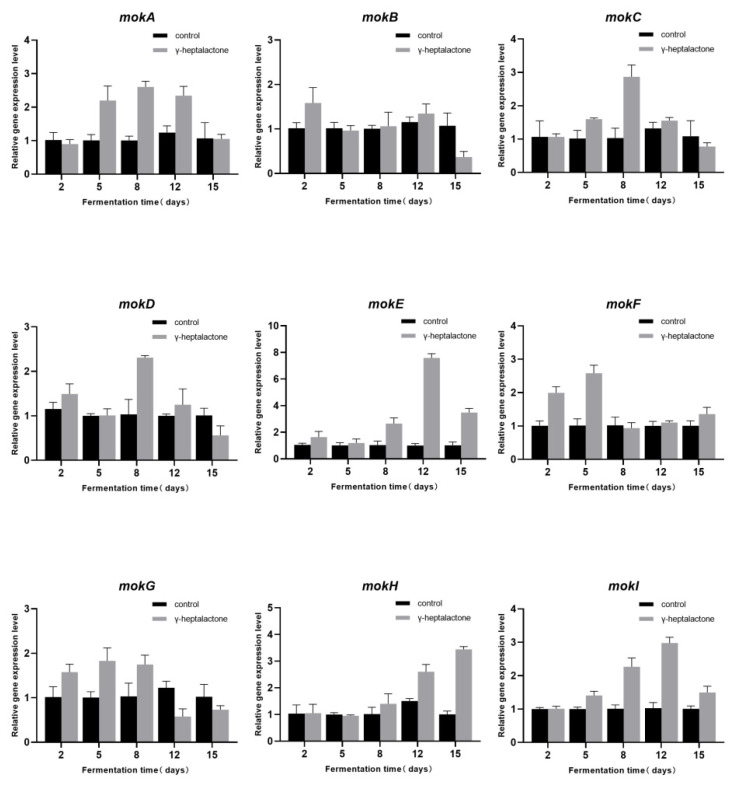
Effect of γ-heptalactone on the relative expression levels of MK biosynthesis genes (*mokA*–*I*).

To investigate the effect of γ-heptalactone on MK metabolism, we measured the expression level of the MK biosynthetic gene cluster (*mokA*–*mokI*) by RT-qPCR(Figure 4). The expression level of *mokB* on day two was 1.56-fold higher than that in the control group. The expression level of *mokF* reached its peak on day 5, which was 2.55-fold higher than the control group. On day 8, the expression levels of *mokC*, *mokD*, *mokE,* and *mokG* genes were significantly up-regulated in the treatment group, which were increased by 2.78-, 2.23-, 7.53-, and 1.82-fold, respectively. The *mokA* and *mokI* genes reached the peak on day 12 and were increased by 2.59- and 2.72-fold, respectively, compared with the control group. The expression of *mokH* gene reached its highest level on day 15, which was 3.42-fold higher than that in the control group.

### 3.5. Effect of γ-Heptalactone on the Mycelial and Spore Morphology of M. purpureus

**Figure 5:**
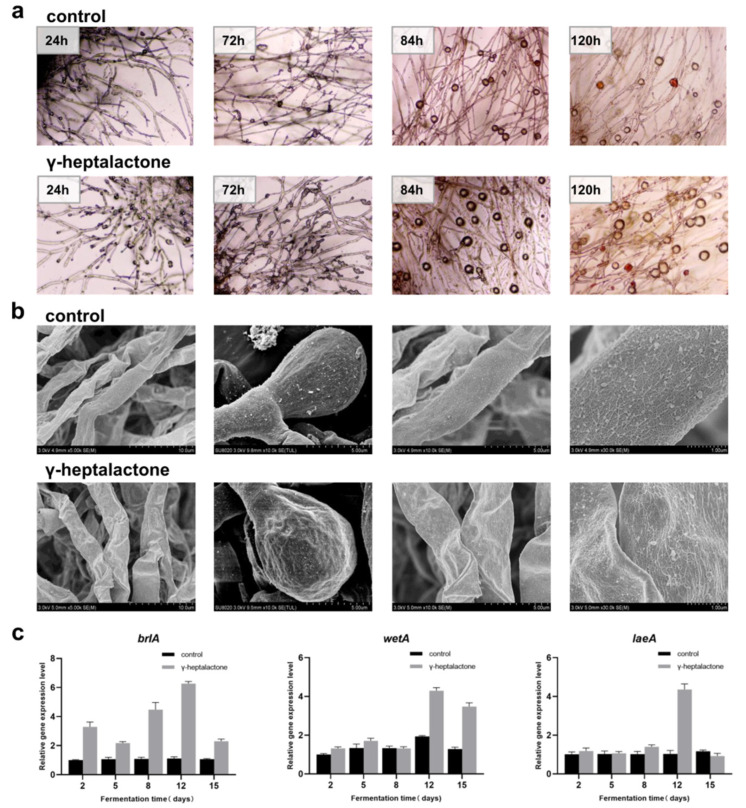
Effect of γ-heptalactone on micromorphology and the expression of asexual development genes of the *M. purpureus* M1. (**a**) Morphology of the *M.* *purpureus* after cultivation on PDA at 30 ℃ for 120 h (at 400× magnifications). (**b**) Scanning electron microscope images of the mycelium and spores at 5000×, 10,000×, and 30,000×. (**c**) Relative expression levels of *laeA*, *brIA*, and *wetA* based on RT-qPCR.

*Monascus purpureus* M1 spore suspension was cultured on PDA (with/without γ-heptalactone) and was observed using a light microscope (at 400× magnifications) (Figure 5a). Mycelial growth and development were dramatically increased after γ-heptalactone treatment, and conidial germination was detected at the end of the mycelium at 24 h. At 72 h, there were many conidia formed, which was much more in the treated group than that in the control group. Ascospore formation was observed at 84 h, and the number of ascospores in the γ-heptalactone-treated group increased compared to the control group. At 120 h, the number of ascospores in the control group was reduced, but many ascospores could still be observed in the treated group.

To observe the structures of *M. purpureus* mycelium and spores, samples were assessed using a scanning electron microscope (SEM; at 5000×, 10,000×, and 30,000× magnifications), as shown in Figure 5b. The mycelial surface with the γ-heptalactone treatment exhibited folds and expansion, whereas the mycelia in the control group were fuller with a smooth surface. The pitting and folding degree of the spores in the γ-heptalactone-treated group were markedly higher than those in the control group. More secretions were accumulated in the treated group than that in the control group. Additionally, RT-qPCR was performed on two central regulators (*brlA* and *wetA*) and a global regulator (*laeA*) in *M. purpuureus M1* to investigate the impact of γ-heptalactone on spores at the transcriptional level. As shown in Figure 5c, the results indicated that after γ-heptalactone treatment, the expression levels of *laeA*, *brIA,* and *wetA* genes were increased by 3.68-, 5.70-, and 2.25-fold, respectively, compared to those in the control.

## 4. Discussion

Lactone molecules, as inductive agents, regulate the cell growth and synthesis of secondary metabolites in *Aspergillus* [51,52]. The addition of butyrolactone I is a potential approach to increase the production of lovastatin in *Aspergillus terreus* [53]. Because the MK biosynthetic pathway in *Monascus* is very similar to the lovastatin biosynthetic pathway in *A. terreus*, we speculate that lactone might be able to improve the production of MK in *M. purpureus* [29]. We selected five lactones for testing and found that both γ-butyrolactone and γ-heptalactone could promote MPs and MK production in *M. purpureus*, with γ-heptalactone having the most pronounced effect. Additionally, the mycelium and spore morphology were also significantly affected by the treatment with γ-heptalactone. This result coincided with Erkaya’s study, which indicated that inductive molecules such as tyrosol and farnesol could increase MPs production [62].

MPs and MK are the best-known secondary metabolites produced by *Monascus* strains, and their biosynthetic pathways have been elucidated [29,56,57]. Treatment with γ-heptalactone was assayed to increase the yields of three MPs (yellow, orange, and red pigments) by 115.70, 141.52, and 100.88%, and reached 67.05, 53.83, and 86.25 U/mL, respectively (Figure 2a–c). The biosynthesis of MPs is regulated by genes encoding various polyketide synthases (PKSs), fatty acid synthases (FASs), dehydrogenases, transporters, and regulatory factors. Therefore, analysis of the expression levels of MPs biosynthesis-related with or without γ-heptalactone treatment was performed [63,64,65]. The results showed that the expression levels of most of the tested genes were significantly upregulated, such as *MpPKS5* (encoding a PKS), *MpFasA2* (encoding a FAS), *mppB* (encoding an acetyltransferase), *mppC*/*G* (encoding oxidoreductases), *mpp7* (encoding an acyltransferase), and *mppR1*/*R2* (encoding regulatory proteins), which increased the yield of MPs (Figure 3). It can be speculated that by upregulating *MpPKS5*, *MpFasA2,* and *mppB*, the relative activities of β-ketoacid and hexoketochromophore enzymes were increased, thus improving the metabolic pathway of the pigment synthesis. In addition, the *mppC, mpp7,* and *mppG* genes were also upregulated, promoting the conversion of orange and yellow pigments.

The maximum increase in MK production was observed when cells were treated with 25 μM γ-heptalactone. The yield of MK reached 187.44 mg/L on day 15, which was 62.38% higher than that in the control (*p* < 0.05). At least nine key genes are associated with MK biosynthesis. In the presence of γ-heptolactone, *mokA*, *mokC*, *mokE*, *mokH,* and *mokI* were significantly affected. MK production in the *mokA*-dissrupted *Monascus* strain has been reported to be completely blocked, indicating the importance of the *mokA* gene in MK biosynthesis [29,32,66]. Meanwhile, the overexpression of *mokC*, *mokE,* and *mokH* significantly affects MK anabolism [60,61]. The *mokI* gene encodes the MokI protein, which functions as an efflux pump [55]. The results pertaining to the *mokI* gene were consistent with those of previous studies; the expression level of *mokI* was much higher in the treated group, implying a significant effect of γ-heptalactone on the anabolism of MK. We predicted that γ-heptalactone might facilitate MK production partly by improving its secretion and reducing the intracellular content.

Previous studies have illustrated the maximal orange and red pigments as 133.77 and 108.02 U/mL, respectively, after 10 days at 30 ℃ with 150 rpm [58]. In our study, γ-heptalactone was assayed to increase the yields of three MPs (yellow, orange, and red pigments) by 115.70, 141.52, and 100.88%, respectively, and reached 67.05, 53.83, and 86.25 U/mL, respectively, at 30 °C, 200 rpm for 2 days, then at 25 °C, 150 rpm for 13 days. The amount of inoculum, carbon source, and glycerol have a great impact on the production of Monascus pigments. Due to the differences in strains, inoculum, carbon source, and culture conditions, there is a gap between the yield of Monascus pigments studied by us and that in the literature.

*Monascus purpureus* M1 is a stable producer of MK; Zhang et al. found that the maximal yield of MK (68.6 mg/L) was observed on day 8 in the original medium, and the maximal yield of red pigment was 40 U/mL [41]. The maximum increase in MK production was observed when treated with 25 μM γ-heptalactone. On day 15, the yield of MK reached 187.44 mg/L, which was 62.38% higher than that in the control (*p* < 0.05). The difference in the optimal inoculation amount required by different fungal strains to reach the highest yield of MPs and MK can be attributed to the composition of the culture medium, growth conditions, environmental stress, and the efficacy of fungal strains.

The apparent increase in secretion on the mycelial surface was also consistent with the genetic results. In our study, the exogenous addition of γ-heptalactone induced mycelial and spore changes, and the upregulation of three genes, *laeA*, *brlA,* and *wetA*, was detected, which is consistent with the findings of a previous study that added butyrolactone to *Aspergillus* [53,67]. In recent years, population induction in filamentous fungi has also been reported, and both fungal morphology and secondary metabolite production are related to the cell population density [7,68,69]. Quorum sensing (QS) exists in a variety of filamentous fungi, and farnesol, tyrosol, lactones, linoleic acid, and oxylipins (oxytetracycline) are among the self-induced quorum sensing molecules (QSMs) that regulate QS behaviors, such as mycobacterial morphology and secondary metabolite alterations [52,70,71]. In *A. terreus*, the addition of butyrolactone I caused a significant increase in lovastatin production and an increase in mycelial tip number and spore production. It was also noted that the addition of butyrolactone I affected the number of spores on apical mycelia, induced morphological and sporulation changes in *A. terreus,* and enhanced secondary metabolite production [52,53]. Moreover, an increase in the hyphal tip numbers and sporulation has been reported for cultures supplemented with butyrolactone I [72,73]. γ-Heptalactone affected the production of penicillin in *A. nidulans* and increased the expression level of the related genes, compared with the untreated control group. This is consistent with previous reports on butyrolactone as QSMs in filamentous fungi [50]. Butyrolactone can affect the production of lovastatin, because it acts as a quorum-sensing molecule in *A. terrestris*. The lactones act as quorum sensing molecules and affect the yield of metabolites through quorum sensing [51,52,53].

In our study, the exogenous addition of γ-heptalactone induced mycelial and spore changes, and the upregulation of three genes, *laeA*, *brlA,* and *wetA*, was detected, which is consistent with the finding in the study of adding butyrolactone I to *Aspergillus* [53]. The effect of γ-heptalactone on the growth and development of the bacterium is presumed to be mediated by LaeA, BrlA, and WetA [74,75]. Similar to that in *Aspergillus*, it has been reported that four proteins, VeA, VelB, VelC, and VosA, together with a global regulator, LaeA, were responsible for the regulation of the hyphal organization and the switch between primary and secondary metabolism [76,77,78]. Spores production is also partly regulated by the LaeA protein. BrlA, AbaA, and WetA are three key regulators that control conidiophore and bud formation, petiole emergence, and conidiophore maturation and spore viability, sequentially [78,79]. Our results showed that γ-heptalactone treatment significantly affected the mycelial development and spore morphology of *Monascus,* and upregulated the expression of *brlA*, *wetA*, and *laeA*, implying a possible effect of γ-heptalactone treatment on the control of cellular differentiation in *Monascus*.

The regulation of secondary metabolite production is thought to be a fundamental feature of QSMs. The addition of QSMs not only affects the morphology of filamentous fungi, but also enhances the synthesis of metabolites. Our study showed that γ-heptalactone can affect the metabolic synthesis of *Monascus* and cause morphological changes, possibly acting in a population-sensing-like manner, and thus might be among the inducing molecules. γ-heptalactone effectively increased the yields of MPs and MK, and regulated the expression of genes related to MPs and MK biosynthesis and metabolism. The underlying mechanism of γ-heptalactone on metabolites might be related to the development of mycelia, which may be associated with QS. This study provides a new strategy to enhance the secondary metabolites of *Monascus*, and, therefore, the detailed mechanism of the effect on the secondary metabolites of *Monascus* needs to be further investigated.

## 5. Conclusions

The γ-heptalactone effectively increased the yields of MPs and MK, as well as the regulation of the gene expression related to them. The underlying mechanism of γ-heptalactone on metabolites might be related to the development of mycelial, which may be associated with QS. This study provides a new strategy to enhance the secondary metabolites of *Monascus*, and, therefore, the detailed mechanism of the effect on the secondary metabolites of *Monascus* needs to be further investigated.

## Figures and Tables

**Table 1:** Primer sequences for the key genes in *M. purpureus* M1.

Genes	Primer sequences (5′to3′)	Length (bp)	Tm Value	PCR Product Length (bp)	Description
*MpPKS5* F	TGTCCGACGAGTTTCTGCAA	20	58	134	NR-PKS
*MpPKS5* R	TATCAACGCTGCTTGGGCAT	20	60	134	
*MpFasA2* F	ATGGATCGCCCGATCTTGTC	20	59	129	FAS subunit alpha
*MpFasA2* R	CTTTGTCGAGTCCGCTGGAT	20	59	129	
*MpFasB2* F	CCTCCAGGGATTACAACCCG	20	58	131	FAS subunit beta
*MpFasB2* R	ATTCAATGCCAGGTGCTCCA	20	58	131	
*mppA* F	TCCCGTTTCTTGGACGTGAG	20	59	132	C-11-ketoreductase
*mppA R*	ACGTGCCATGGTTCTGTCTT	20	59	132	
*mppB* F	CGTCTCGCCCGATAACTTCA	20	60	108	acyltransferase
*mppB R*	TTGACAGACGGGTCGAAGTC	20	58	108	
*mppC* F	CAGTCCTCGTCCCTTCCAGT	20	60	137	NADPH-dependent oxidoreductase
*mppC* R	CCACGGTGAAGGATGTCGAG	20	58	137	
*mppD* F	TCAACACGGGAGATGCTGTC	20	62	140	serine hydrolase
*mppD* R	GCCAAAGGACAGGAGCAGAT	20	63	140	
*mppE* F	CTTCCCGATGCCGTTGTGAT	20	60	99	enoyl reductase
*mppE* R	GTCTCGTGGATCATCTCGT	19	60	99	
*mppG* F	TCAACACGGGAGATGCTGTC	20	56	140	FAD-dependent oxidoreductase
*mppG R*	GCCAAAGGACAGGAGCAGAT	20	59	140	
*mpp7* F	ATCGTCGGATCAGCGTCAC	19	59	148	acetylatransferase
*mpp7* R	CGGCTGTTATAGGGTGGC	18	57	148	
*mppR1* F	TCTGCAGTATGCCATGTGGG	20	59	123	transcription factor
*mppR1* R	ATGGCACCGTCACTTAGCTC	20	55	123	
*mppR2* F	ACGAAACCCTCCATGACACC	20	59	138	transcription factor
*mppR2* R	TGCAGACAGCCTTGTGGTAG	20	59	138	
*mokA* F	GACCTCGGTCATCTTGGC	18	57	78	polyketide synthase
*mokA* R	TTGTTCCAAGCGGTCTTC	18	54	78	
*mokB* F	AAACATCGTCACCAGTCT	18	53	78	polyketide synthase
*mokB* R	CTAAGTCGGGCATCTACC	18	53	78	
*mokC* F	CAAGCTGCGAAATACACCAAGCCTC	25	62	80	P450 monooxygenase
*mokC R*	AGCCGTGTGCCATTCCTTGTTGTCC	25	60	80	
*mokD* F	TTCATCTGCTGCTGGTAT	18	53	92	oxidoreductase
*mokD* R	AACTTCTCACCGTCAATG	18	52	92	
*mokE* F	ATCGCAGGTCACGCACATCCAAGTC	25	65	221	dehydrogenase
*mokE* R	GTAAAGGCAGCCCGAGCAGCTTCAT	25	65	221	
*mokF* F	GAGATCATAGTGGCCGACTGAA	22	60	190	transesterase
*mokF* R	ACCGTCTCATCCAACCTCACGA	22	61	190	
*mokG* F	CCAGGTAACCAACGGATTA	19	51	82	HMG-CoA reductase
*mokG* R	GATCAGAGCAGTCACCAG	18	54	82	
*mokH* F	CAGGAAATCTGGACTTACCCCATTG	25	58	123	transcription factor
*mokH* R	TGTTGGATTGTTGTTGGAGATATAC	25	55	123	
*mokI* F	CAGGAAATCTGGACTTACCCCATTG	20	60	135	efflux pump
*mokI* R	TGTTGGATTGTTGTTGGAGATATAC	18	57	135	
*laeA* F	ACTCGTAGCGGATGTAAGA	19	55	105	global regulator
*laeA* R	CCGTGCTTGGTAGATGTG	18	55	105	
*brIA* F	ATGTCAGGGTGGCGTGAAGT	20	60	187	asexual development
*brIA* R	CCTGAACTGTACCTGCTTGAT	21	56	187	
*wetA* F	ATGTGTTATATTCCCCGGGA	20	60	174	asexual development
*wetA* R	TTAGCAGAGTGCGGCCTCGAG	21	62	174	
*GAPDH* F	CCGTATTGTCTTCCGTAAC	19	55	114	Reference gene
*GAPDH* R	GTGGGTGCTGTCATACTTG	19	56	114	

## Data Availability

The data in this study are available in this article.
